# Exploratory cost-effectiveness analysis of cardiac resynchronization therapy with systematic device optimization *vs*. standard (non-systematic) optimization: a multinational economic evaluation

**DOI:** 10.1186/s13561-015-0057-3

**Published:** 2015-07-11

**Authors:** Kurt Banz, Peter Paul Delnoy, Jean Renaud Billuart

**Affiliations:** 1 OUTCOMES International Ltd, Malzgasse 9 CH- 4052, Basel, Switzerland; 2 Isala Klinieken, Zwolle, Netherlands; 3 Sorin CRM, Clamart, France

**Keywords:** Cardiac resynchronization therapy (CRT), Cost effectiveness, Optimization of CRT therapy

## Abstract

**Background:**

Recent studies provide evidence of improved clinical benefits associated with cardiac resynchronization therapy (CRT) optimization. Our analysis explores the cost-effectiveness of systematically optimized (SO, 3 times a year) *vs*. non-systematically optimized (NSO, less than 3 times a year) CRT, whatever the echo optimization method used (manual or SonR® automatic).

A longitudinal cohort model was developed to predict clinical and economic outcomes for SO *vs*. NSO strategies over 5 years. The analysis was performed from the payer perspective. Data from CLEAR study post-hoc analysis was used with 199 pts with CRT pacemaker (CRT-P). The main economic outcome measure was incremental cost-effectiveness (ICER) expressed as cost per Quality Adjusted Life Years (QALY) gained. To assess the impact of data uncertainty, a sensitivity analysis was performed. The model also predicts outcomes for the two optimization strategies for CRT-D therapy *vs.* optimal medical treatment (OPT).

**Results:**

At 1 year, ICERs for SO CRT vs. NSO CRT-P range between € 22,226 (Spain) and € 26,977 (Italy). Therefore, on the basis of a Willingness-To-Pay of €30,000 per QALY, the SO method develops into a cost effective strategy from 1 year, onwards. These favorable outcomes are supported by the sensitivity analysis. Systematic optimization of CRT-D might also improve the cost-effectiveness of this device therapy by 27 % to 30 % dependent on the country analyzed, at 5 years.

**Conclusions:**

Our economic evaluation shows promising health economic benefits associated with SO CRT. These preliminary findings need further confirmation.

**Electronic supplementary material:**

The online version of this article (doi:10.1186/s13561-015-0057-3) contains supplementary material, which is available to authorized users.

## Background

Heart failure (HF) is a growing worldwide public health issue and constitutes enormous medical, social and economic problems. In Europe, 1-2 % of the general population is affected by HF with a rapid increase in prevalence for the older age groups [1, 2]. Around 3.5 million people are newly diagnosed with HF every year [3]. Morbidity and mortality caused by this chronic and progressive disease is substantial with observed death rates after one year of diagnosis as high as 40 % without adequate therapy [4–6]. Due to its significant frequency coupled with the high morbidity and mortality, medical resource utilization devoted to the care of patients with HF is considerable, resulting in a high financial burden to healthcare payers. For numerous developed countries the management of HF has been shown to account for at least 1-2 % of total health care expenditures [7].

For the treatment of patients with advanced HF and evidence of intraventricular conduction delay who are refractory to optimal pharmacological therapy, cardiac resynchronization therapy (CRT) is the established standard treatment. The clinical benefits conferred by CRT in selected patient populations with advanced HF and cardiac dyssynchrony have been unequivocally demonstrated in numerous controlled trials [8]. Benefits include improvements in the clinical status and functional capacity, cardiac remodeling, reduction in the frequency of hospitalizations from HF, prolongation of survival, and an increase in the quality of life. However, about 30-40 % of recipients of CRT may not improve in their clinical status based on current selection criteria [8]. This significant non-response rate can be ascribed to a variety of reasons including suboptimal patient selection, inappropriate lead positioning, or suboptimal CRT programming (device optimization) [9]. It is now recognized that optimal follow-up of patients with CRT is crucial for ensuring that patients will derive maximum benefit from this therapy [10]. Continuous optimization of both atrioventricular delay (AVD) and interventricular delay (VVD) has been advocated as technique to improve responder rates [11, 12].

The meta-analysis done by Auger et al. [13] clearly emphasizes the ambiguity of the conclusions of previous studies related to AV-VV optimization. Among the method described by the author, echo-optimization method (either done manually or automatically) shows much more promising outcomes as compared with other methods. One of the difficulties related to echo-optimization is ability to replicate consistently echoes. Previous studies have illustrated that resource constrains (availability of echo staff and time) impede doctors from doing AV-VV optimization even if guidelines indicate that optimization is an alternative for non-responders [14].

In addition, echocardiography is often inadequately reimbursed. Consequently, CRT is usually not systematically optimized in routine clinical practice [15, 16]. This has been demonstrated by a recent survey which discovered that echo-optimization is performed infrequently whether at the time of system implant, or during follow-up [15]. In an effort to overcome these challenges, automated methods adapting CRT delivery according to patients’ changing needs are being developed, allowing a more efficient approach to CRT optimization. However, earlier non-echo CRT optimization algorithms have produced inconclusive results in terms of clinical benefits, calling for new methods [15]. The CLEAR (Clinical Evaluation on Advanced Resynchronization, Clinicaltrial.gov registration number: NCT00658203) pilot study is the first trial showing a trend towards better outcomes associated with automated AVD and VVD optimization employing a hemodynamic sensor that records the peak endocardial acceleration (SonR) signal [12]. The SonR signal is correlated to hemodynamic measures [17] and allows automatic echo optimization according to a method similar to the RITTER formula. In a recent post-hoc analysis of patients having completed the CLEAR pilot study, clinical benefits conferred by systematic CRT optimization in comparison to non-systematic optimization over a follow-up period of one year have been reported [18].

Due to the significant up-front cost, there has been great interest in evaluations of whether CRT therapy, potentially combined with a defibrillator (CRT-D), is cost-effective when compared to optimal pharmacological treatment alone [19]. Yet, none of these economic studies had specifically addressed the consequences associated with different follow-up CRT optimization strategies. Since first study data showing the beneficial impact of optimization on clinical outcomes are emerging, our exploratory analysis therefore sought to assess the economic value of CRT implantation with systematic optimization (SO, 3 times a year) in comparison to standard (non-systematic, NSO) CRT optimization (less than 3 times a year), in five European countries (Germany, France, Italy, Spain, and the UK).

## Methods

### Longitudinal deterministic model

A longitudinal deterministic cohort model was developed to evaluate the clinical and economic outcomes for a group of CRT recipients with systematic CRT optimization subsequently to device implantation *vs*. a group with standard (NSO) optimization, whatever the method used (echo-based or using the SonR system). A healthcare (payer) perspective was considered for the economic analysis. The model allows predicting outcomes (death, HF hospitalization, NYHA class distribution) up to 5 years with distinctive analysis time points at 1, 2, 3 and 4 years. The model predicts cumulative total costs and health outcomes, i.e., quality adjusted life years (QALYs) accrued over time, and the incremental cost-effectiveness ratio (ICER) of SO *vs*. NSO CRT-P. Sensitivity analyses were performed to assess the impact of variations in assumptions of key model parameters on the economic outcome. Furthermore, a hypothetical scenario analysis was executed to estimate the potential cost-effectiveness of SO and NSO CRT-D recipients in comparison to patients treated with optimal pharmacological therapy (OPT) alone.

### Data sources

For our analysis, data from a post-hoc analysis of the CLEAR study was used, it included all patients (n = 199) who had a successful CRT pacemaker implantation and who completed the 1-year follow-up [18]. The group of patients with SO comprised 66 individuals who were systematically optimized at all 3 pre-specified visits (post-hospitalization, 3 and 6 months), whereas the NSO group included 133 patients who were optimized less than 3 times a year, regardless of the echo optimization method used (either manual- or by the SonR echo based system). Therefore, mortality, hospitalization for HF rates and distributions of NYHA functional classes were retrieved from the CLEAR post-hoc analysis, for the 2 groups, at each time point up to one year (Table 1).

**Table 1:** Base case clinical model input data assumptions

Model variable									Source
	Month 1	Month 3	Month 6	Year1	Year 2	Year 3	Year 4	Year 5	
All-cause mortality^a^									
Systematic CRT optimization	0.0 %	0.0 %	3.0 %	6.8 %	17.8 %^a^	25.8 %	33.8 %	40.8 %	[18, 20], assumption
Standard CRT optimization	1.5 %	3.0 %	6.8 %	14.3 %	25.3 %	33.3 %	41.3 %	48.3 %	[18, 20]
HF hospitalization^b^									
Systematic CRT optimization	0.0 %	3.0 %	7.6 %	12.2 %	22.5 %	32.7 %	43.0 %	52.4 %	[18], assumption
Standard CRT optimization	3.8 %	8.3 %	13.5 %	23.3 %	42.9 %	62.5 %	82.1 %	100.0 %	[18], assumption
Mean NYHA class	Baseline	Month 3	Month 6	Year 1	Year 2	Year 3	Year 4	Year 5	
Systematic CRT optimization									[18], assumption
NYHA I	0 %	16.9 %	26.6 %	22.2 %	22.2 %	22.2 %	22.2 %	22.2 %	
NYHA II	10.9 %	75.4 %	65.6 %	63.5 %	63.5 %	63.5 %	63.5 %	63.5 %	
NYHA III	87.5 %	7.7 %	7.8 %	14.3 %	14.3 %	14.3 %	14.3 %	14.3 %	
NYHA IV	1.6 %	0 %	0 %	0 %	0 %	0 %	0 %	0 %	
Standard CRT optimization									[18], assumption
NYHA I	0 %	8.5 %	18.7 %	13.8 %	13.8 %	13.8 %	13.8 %	13.8 %	
NYHA II	6.9 %	62.7 %	58.0 %	56.9 %	56.9 %	56.9 %	56.9 %	56.9 %	
NYHA III	86.1 %	28.0 %	20.5 %	27.5 %	27.5 %	27.5 %	27.5 %	27.5 %	
NYHA IV	6.9 %	0.8 %	2.7 %	1.8 %	1.8 %	1.8 %	1.8 %	1.8 %	
Health utilities by NYHA class									
NYHA class I	0.815								[19]
NYHA class II	0.720								[19]
NYHA class III	0.590								[19]
NYHA class IV	0.508								[19]
Death	0								[19]

^a^In accordance with the conservative approach adopted for the present analysis, the incremental increase in mortality after 1 year for the systematic CRT optimization group was assumed to be identical to that applied to the standard CRT optimization group, i.e. benefits of CRT optimization on mortality were assumed to cease after one year of follow-up

^b^Values are presented as cumulative probability

### Base case

Individual CRT optimization regimens can be simulated. As types of optimization procedures performed can vary and related time needs and tariffs can differ, the model permits a choice between a routine consultation, a consultation including echo control, or a consultation with echo optimization. Model assumptions on the CRT optimization schedules for the 2 groups are in accordance with the optimization procedures reported in the CLEAR post-hoc study (Additional file 1: Table A).

**Fig. 1:**
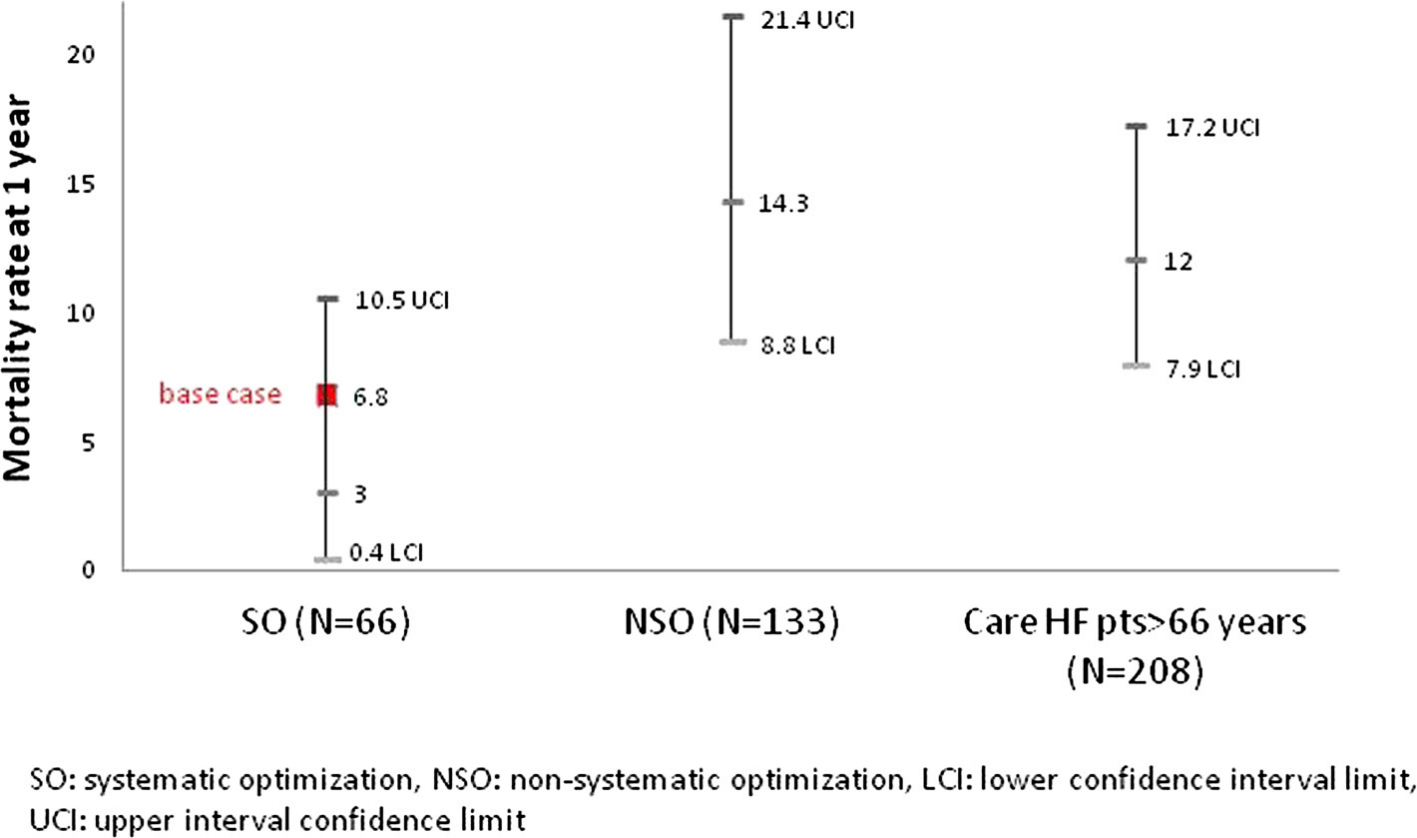
Death rate with the value of the average between the actual rate and the upper confidence interval limit in the CLEAR study and CARE-HF study (patients >66 year-old), at 1 year

As the CLEAR study provides data for a follow-up period of one year only [18], assumptions were employed to inform the model for the time periods from 2 years and beyond. Considering the exploratory purpose of this analysis, conservative assumptions were selected as compared with actual rates. In order to draw reasonable assumptions beyond 1 year, mortality and HF hospitalization rates at 1 year in the SO group were adjusted as an average between the actual rate and the upper confidence interval limit (CI) from the CLEAR post-hoc analysis (Fig. 1). Beyond 1 year, the incremental increase in mortality was assumed to be identical to that applied to the NSO group, which has been derived from CARE-HF long-term CRT outcome study published by Cleland and co-workers [20]. As the CLEAR study population is older than the CARE-HF population, assumptions relative to mortality for the NSO arm refer to long-term CARE-HF clinical data for patients above 66 years [20]. This is considered a conservative assumption, implying that the demonstrated survival benefits related to SO which has been observed during the first year will not continue thereafter (Table 1).

With regard to assumptions on the probability of HF hospitalizations after one year for patients with NSO, a linear increase was assumed based on the incremental rate of hospitalization between 6 and 12 months observed for this group in the CLEAR post-hoc study. From an economic point of view, hospital admissions caused by acute episodes of HF are of particular importance for the analysis, as these events are frequent and costly. This is also apparent from the CLEAR study, particularly as concerns the NSO group, which shows a substantial percentage of patients requiring hospitalization for acute HF symptoms in the first year after implantation. In contrast, SO leads to a significant decrease in the frequency of rehospitalizations. One year post-hoc study data further indicated that for each hospitalized patient, the mean number of admissions was 1.4 in both groups, and was thus also factored into our economic model. In order to validate this assumption, information on the probability of HF hospitalization presented by Anand and co-workers was reviewed [21]. A comparison of the frequency of HF hospital admissions (considering recurrent admissions) within the first year after CRT implantation showed an excellent fit between the CLEAR study and this reference. This analysis is in line with a recent publication from Maneikiene and co-authors relative to the outcome of cardiac resynchronization therapy in older patients [22]. In addition, the increase in the cumulative probability of HF hospitalization with ongoing follow-up time (up to around 2.5 years) as observed by Anand showed a strong tendency for a linear correlation, which substantiates the longer-term assumptions applied to our model.

In terms of the frequency of HF hospitalizations after one year for the systematic optimization group, the same relative increase in the probability of HF events as assumed for the SO group was applied. This approach accounts for expected long-term benefits of SO on re-hospitalizations (Table 1).

Regarding the NYHA class, the distribution observed at the one year follow-up of the CLEAR post-hoc study was assumed to remain unchanged up to 5 years (Table 1).

### Quality-adjusted life-years (QALYs)

Patients’ status in the SO group was associated with more frequent transitions towards lower NYHA functional classes than observed for patients with non-systematic optimization according to the CLEAR post-hoc analysis. This beneficial effect led to a decrease in persisting symptoms of cardiac insufficiency and consequently improves the quality of life (QoL) of HF patients. The majority of previous economic model analyses evaluating the effects of CRT have also considered shifts in QoL, typically by accounting for changes in distributions of subjects in the compared groups across the four NYHA classes over time and assigning a utility weight to each NYHA class in order to compute QALYs [19, 23]. Therefore, the model calculated the cumulative number of QALYs in each group, by assigning health utilities to each NYHA functional class patients’ distribution and death rate (Table 1).

### Economic data

Country-specific tariffs were allocated to medical resource use items, including CRT implantation, hospital readmissions due to HF episodes or complications, and follow-up physician visits including diagnostic examinations associated with CRT (Table 2). Pharmacological treatment costs were based on prices from national drug compendiums. All unit cost data used to inform the model were adjusted to fiscal year 2012. Half-cycle correction methods were applied to the computation of both costs and effectiveness results. Future costs and health benefits were discounted according to recommended rates as specified in national health economic guidelines. Table 2 summarizes the economic input data for the different economic model variables and for the base case analysis involving patients with CRT-P implantation. For the purpose of our analysis, an extra expense of €2,000 was assumed for additional optimization for SO (echo- or SonR-based optimization) *vs*. NSO. This is consistent with previous investigations identifying lack of resource as one of the key difficulties in conducting more systematically echo optimization [16]. In terms of the threshold determining cost-effectiveness, a commonly used willingness-to-pay (WTP) threshold of £ 25,000 (€ 30,000) as recommended by the National Institute of Health and Care Excellence (NICE) was employed.

**Table 2:** Base case economic model input data assumptions

Model variable	Germany	France	Spain	Italy	UK
Tariff for CRT-P implantation^a^	€ 11.924	€ 9.412	€ 10,791	€ 8,921	£ 8,281
Actual cost of CRT	€ 12,326	€ 9,280	€ 11,121	€ 8,455	£ 6,474
Cost of CRT procedure					
CRT-P device incl. leads	€ 5,569	€ 5,816	€ 5,428	€ 3,950	£ 3,411
Other costs^b^	€ 3,169	€ 1,654	NA	€ 718	£ 1,555
Premium for sensor lead^c^	€ 2,000	€ 2,000	€ 2,000	€ 2,000	£ 1,665
Unit cost follow-up HF hospitalization^d^	€ 2,328	€ 3,577	€ 3,364	€ 3,052	£ 3,411
Average monthly cost of optimal drug therapy^e^	€ 59	€ 18	€ 18	€ 18	£ 22
Tariff for CRT optimization services					
Routine cardiology consultation	€ 28	€ 61	€ 45	€ 21	£ 62
Consultation with echocardiographic control	€ 28	€ 96	€ 79	€ 52	£ 86
Device optimization by echocardiography	€ 73	€ 96	€ 147	€ 52	£ 120
Actual costs for CRT optimization services^f^					
Routine cardiology consultation	€ 24	€ 28	€ 19	€ 17	£ 17
Consultation with echocardiographic control	€ 62	€ 70	€ 48	€ 36	£ 43
Device optimization by echocardiography	€ 147	€ 169	€ 115	€ 87	£ 101
Discount rate					
Costs	3.0 %	3.0 %	3.0 %	3.0 %	3.5 %
Benefits	3.0 %	3.0 %	3.0 %	3.0 %	3.5 %

^a^Weighted tariff taking into account the relative frequency of implantations by DRG severity category and corresponding tariffs (applies if more than one DRG for CRT is reported in the DRG catalogue)

^b^E.g. cost for personnel involved in CRT implantation, diagnostic examinations, disposables/consumables, medication, overhead costs

^c^Assumption as there is currently no information on the extra cost for the sensor lead available; this expense was applied to the systematic optimization group only and also taken into account (conservative approach) for the analysis performed from the perspective of the healthcare payer although the current DRG tariff for CRT would currently include this expense

^d^Per stay

^e^For Germany based on reference [26]; for the UK based on reference [27]; for remaining countries based on own assumptions

^f^Computed from the estimated duration of optimization service and hourly cost of personnel involved in the optimization procedures (CRT and/or echo specialist)

### Sensitivity analyses

To assess the impact of data uncertainty on the economic outcomes of the base case analysis, univariate and probabilistic sensitivity analyses were performed, considering the model parameters and assumptions summarized in the Additional file 1: Table B. Such a sensitivity analysis is important to assess the robustness of results and conclusions derived from the economic analysis.

### Exploratory CRT-D analysis

An additional scenario analysis was performed to assess the cost-effectiveness of both SO and NSO CRT-D *vs*. optimal pharmacological treatment alone (OPT). For this assessment, the incidence of follow-up events observed in the CLEAR post-hoc study (in which a CRT device without a defibrillator was implanted) were recalculated on the basis of exclusion of those patients from the re-analysis who have died for reasons of sudden cardiac death. This event has been witnessed in 1 patient randomized to the systematic optimization group and 3 patients randomized to the NSO group. This approach suggests that sudden cardiac deaths would have been prevented if CRT-D instead of CRT-P devices would have been used. We assumed no additional benefit attributable to ICD as the frequency of other follow-up events has been reported to be similar for CRT-P and CRT-D patients. Economic input data were adjusted where appropriate to be consistent with the CRT-D setting. Furthermore, a hypothetical scenario analysis was executed to estimate the potential cost-effectiveness of systematic and standard optimization in CRT-D recipients in comparison to patients treated with OPT. To inform the OPT arm of our model, results from the CARE-HF study (patients >66 years-old) were used as assumptions for the cumulative percentage of patients experiencing death and hospital admission due to HF over the first two years [20]. For subsequent analysis time points, annual increases in the percentage of these two main events as observed in the CARF-HF study between one and two years were applied [20]. NYHA distribution for OPT patients was assumed to be identical to that applied to CRT-D patients at baseline and was assumed to remain unchanged over the 5 year follow-up and the average number of hospitalizations per hospitalized patient was adjusted to 2.3 admissions (1.4 for CRT recipients) based on evidence from randomized trials.

## Results

Table 3 summarizes discounted results of the exploratory base case analysis performed from the perspective of the healthcare payer by analyzed time point and by country for patients with CRT implantation. According to these findings, systematic CRT-P device optimization leads to a survival benefit and improved quality-of-life as compared to standard (non-systematic) device optimization. By the end of the first year, systematic device optimization results in a gain of 0.07 QALYs per patient, whereas this benefit increased up to 0.33 QALYs by year 5. With regard to total costs, these remain higher during the early follow-up period for SO, mainly caused by the consideration of a hypothetical extra cost for additional procedure (either a systematic echo procedure or implantation of the SonR sensor) (€ 2,000 or £ 1,665). With ongoing follow-up time, however, these additional costs can partially be offset in the evaluated countries due to savings attributable to prevented HF hospitalizations. This evolution of incremental costs and health benefits returns favorable ICERs (cost per QALY) already at the one year follow-up for all five countries, ranging between € 22,226 (Spain) and € 26,977 (Italy).

**Table 3:** Base case results by treatment group (systematic *vs.* standard CRT-P optimization) from the perspective of the healthcare payer (values represent discounted average per-patient outcomes)

Country	1 year	2 years	3 years	4 years	5 years
Germany					
Total cost – systematic optimization group	€ 15,120	€ 16,143	€ 17,063	€ 17,897	€ 18,625
Total cost – standard optimization group	€ 13,532	€ 14,769	€ 15,896	€ 16,930	€ 17,832
Increment (systematic *vs*. standard)	€ 1,588	€ 1,375	€ 1,168	€ 967	€ 793
Total QALYs – systematic optimization group	0.71	1.32	1.85	2.32	2.72
Total QALYs – standard optimization group	0.64	1.19	1.65	2.05	2.39
Increment (systematic *vs.* standard)	0.07	0.13	0.21	0.27	0.33
ICER^a^	€ 26,973	€ 10,224	€ 5,690	€ 3,556	€ 2 371
France^b^					
Incremental total cost (systematic *vs.* standard)	€ 1,357	€ 971	€ 596	€ 232	€ −88
Incremental total QALYs (systematic *vs*. standard)	0.07	0.13	0.21	0.27	0.33
ICER	€ 23,053	€ 7,222	€ 2,904	€ 853	Dominant
Spain^b^					
Incremental total cost (systematic vs. standard)	€ 1,309	€ 943	€ 587	€ 242	€ −61
Incremental total QALYs (systematic vs. standard)	0.07	0.13	0.21	0.27	0.33
ICER	€ 22,226	€ 7,010	€ 2,862	€ 892	Dominant
Italy^b^					
Incremental total cost (systematic vs. standard)	€ 1,588	€ 1,333	€ 1,076	€ 820	€ 594
Incremental total QALYs (systematic vs. standard)	0.07	0.13	0.21	0.27	0.33
ICER	€ 26,977	€ 9,912	€ 5,244	€ 3,017	€ 1 775
UK^b^					
Incremental total cost (systematic vs. standard)	£ 1,224	£ 993	£ 770	£ 554	£ 367
Incremental total QALYs (systematic vs. standard)	0.07	0.13	0.21	0.27	0.33
ICER	£ 20,787	£ 7,405	£ 3,771	£ 2,055	£ 1,109

^a^The ICER is expressed as cost per QALY gained

^b^Only incremental outcomes and ICERs are tabulated for these countries

**Fig. 2:**
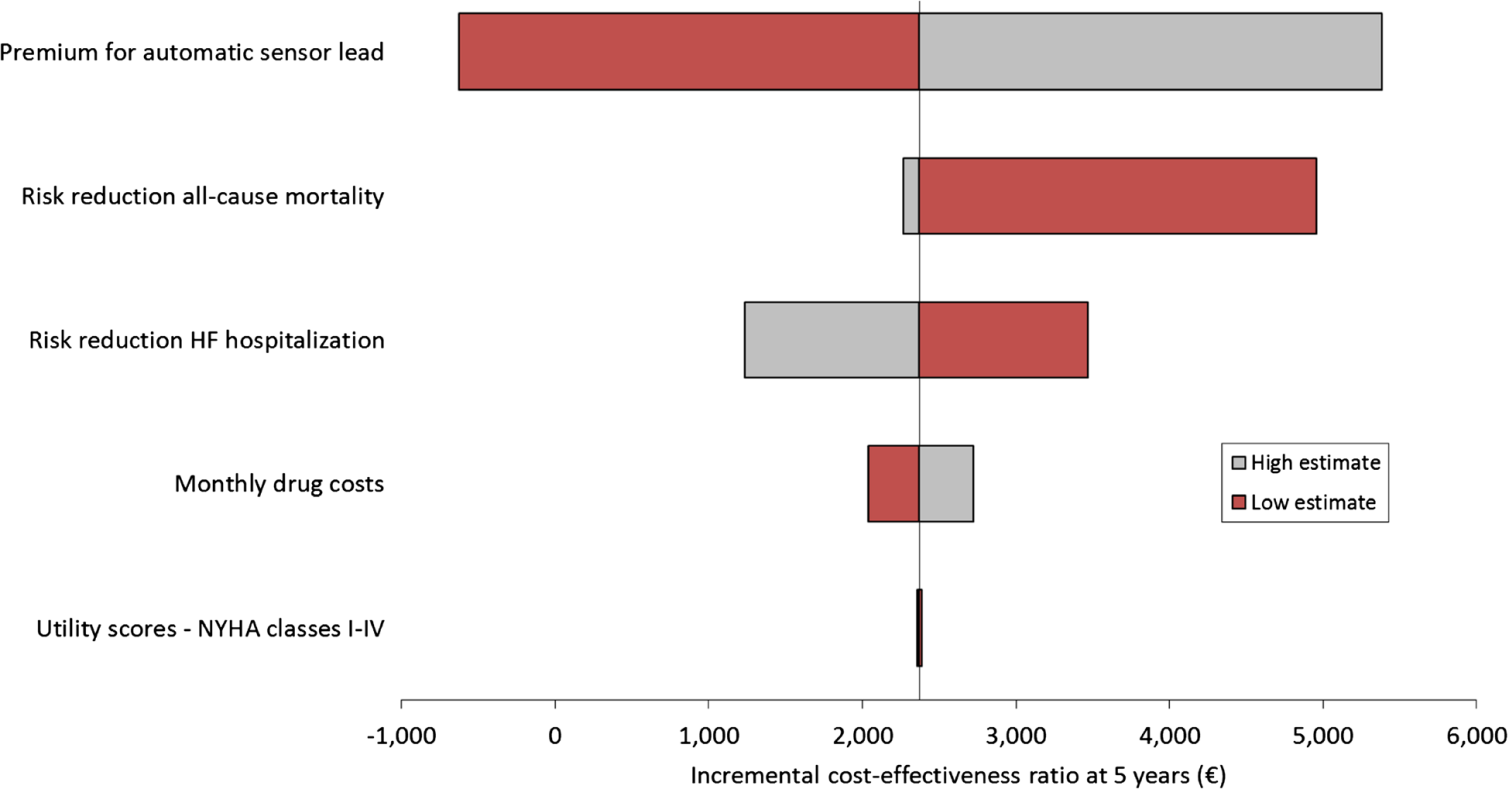
One-way sensitivity analysis illustrating the impact of main model variables on the incremental cost-effectiveness ratio for the 5-year follow-up time horizon (healthcare payer perspective, Germany)

**Fig. 3:**
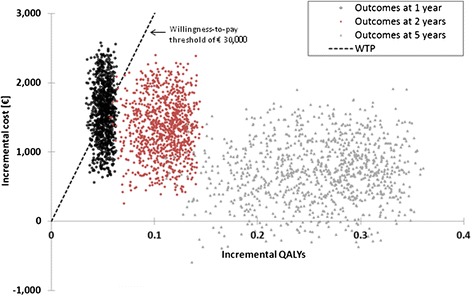
Scatterplot illustrating incremental costs versus incremental benefits (QALYs) for a 1-year, 2-year, and 5-year follow-up time horizon (n = 1,000 simulations, healthcare payer perspective, Germany)

**Fig. 4:**
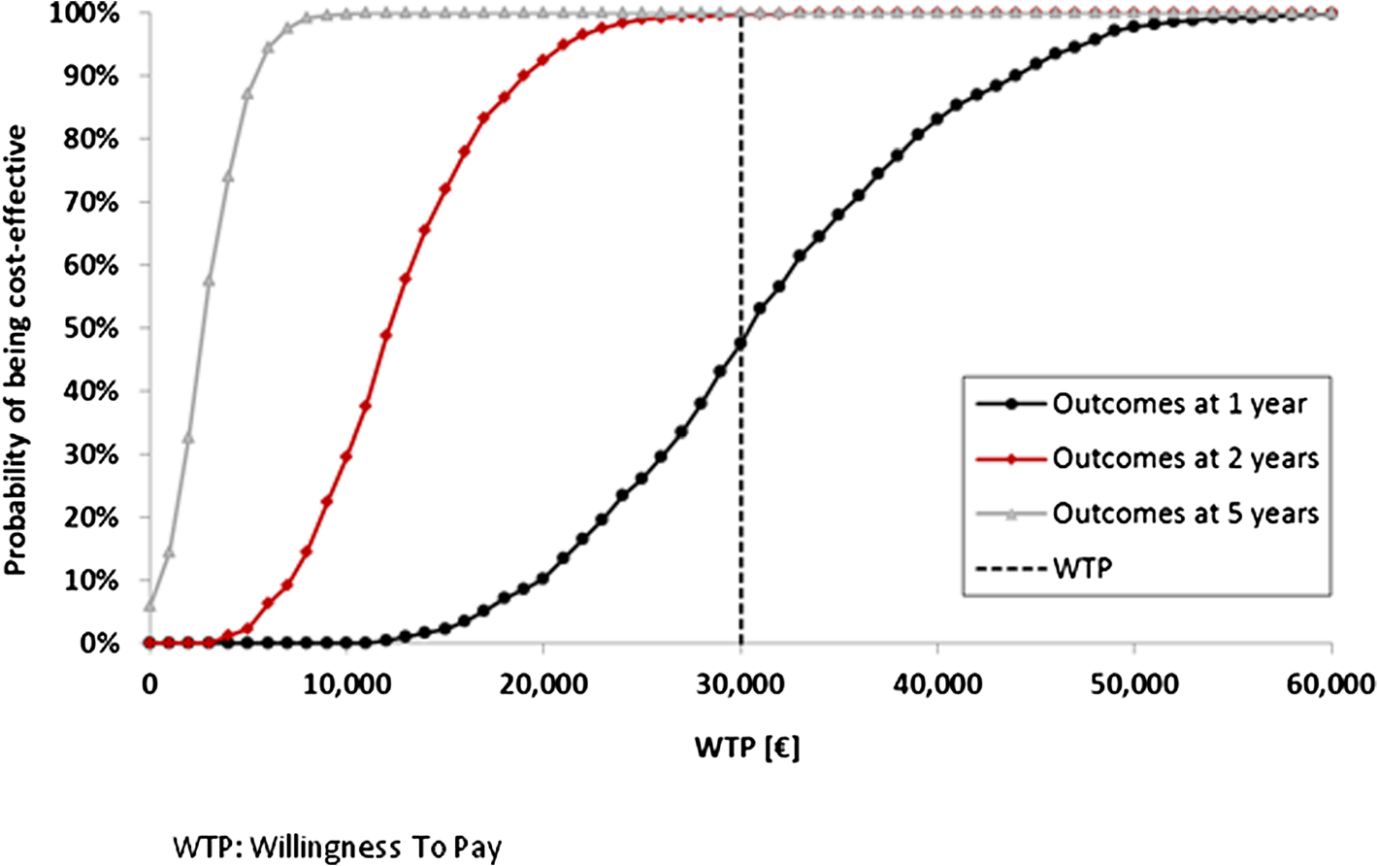
Cost-effectiveness acceptability curve with results produced for a 1-year, 2-year, and 5-year follow-up time horizon (n = 1,000 simulations, healthcare payer perspective, Germany)

By applying the low and high estimates for main model variables (Additional file 1: Table B, example for Germany), the accomplished one-way sensitivity analysis indicated the incremental expense for the automatic sensor lead taken into account for the SO group to be the parameter with the most distinct impact on the incremental cost-effectiveness ratio (ICER) at the 5-year analysis time horizon as illustrated in Fig. 2. Further variables with sizable effects on the ICER include the risk reduction for all-cause mortality (in the worst case, a zero risk reduction on mortality was considered for systematic CRT optimization) and for HF hospitalization ascribed to the SO group. In contrast, variations in the discount rates for costs and benefits were found to affect the ICERs only marginally. The probabilistic sensitivity analysis performed in addition substantiated the validity of the favorable base case results. For this second sensitivity analysis, 1,000 simulations were performed in which values of all selected key model variables were changed simultaneously (Additional file 1: Table B). The findings resulting from the probabilistic sensitivity analysis confirmed the robustness of the base case results as shown in Figs. 3 and 4. Of the 1,000 simulations performed, 47.6 % of ICERs computed for the 1 year follow-up time point were found to be lower than the specified willingness-to-pay threshold (WTP) of €30,000. Already after 2 years, this percentage increases to 99.9 % and after 5 years all computed ICERs were below the specified WTP.

### Exploratory CRT-D analysis

Extrapolating the clinical effects of systematic CRT-P optimization to a hypothetical CRT-D setting, cost-effectiveness results that were almost equivalent to those produced for CRT-P patients were predicted by our model (comparison of systematic vs. standard CRT-D optimization). Systematic optimization might contribute to improve the CRT-D cost-effectiveness (ratio of systematic CRT-D *vs*. OPT and standard CRT-D *vs*. OPT) by 27 % to 30 % depending on the country analyzed (Table 4).

**Table 4:** Cost-effectiveness of systematic CRT-D optimization versus optimal pharmacological therapy (OPT) from the perspective of the healthcare payer

	ICER at 5 years^a^	ICER at 5 years^a^	Difference
	Systematic CRT-D optimization *vs*. OPT	Standard CRT-D optimization *vs.* OPT	Systematic *vs*. Standard
*Country*			
Germany	€ 16,359	€ 22,327	-27 %
France	€ 16,052	€ 23,019	-30 %
Spain	€ 24,440	€ 34,936	-30 %
Italy	€ 15,077	€ 20,738	-27 %
UK	£ 14,950	£ 20,861	-28 %

^a^ICERs are expressed as cost per QALY gained

## Discussion

Despite the evidence-based clinical benefit of CRT, about one third of CRT patients are commonly considered non-responders. One variable that may influence therapy response is the quality of follow-up device optimization [11]. However, the debate on the need for routine, systematic AVD and VVD optimization in all patients undergoing CRT remains controversial [13, 24]. Furthermore, first guidelines on the optimization of CRT in routine clinical practice have been published only very recently [24], which may be another reason for the suboptimal follow-up programming of CRT devices reported by a recent international survey [16]. Main causes for suboptimal optimization were time constraints of medical specialists involved in the management of patients with CRT. This situation calls for less time-consuming optimization methods. Therefore, CRT systems which facilitate follow-up device optimization are being developed and clinically evaluated [13]. For the first time to our knowledge, the post-hoc analysis of the CLEAR pilot study provided preliminary evidence of superior clinical outcomes in terms of reduced mortality and less HF hospitalizations for patients with SO compared to standard practice (NSO) device optimization over a follow-up of one year [18].

The economic consequences pertaining to different follow-up CRT optimization schedules have not been investigated to date except for a budget impact analysis from the perspective of the United States Medicare payment setting which predict savings to occur with automatic CRT devices [13]. We therefore sought to conduct an exploratory cost-effectiveness analysis based on outcome data from the CLEAR post-hoc analysis, complemented by assumptions for the analysis time period between one and 5 years. Whereas identical clinical assumptions were used as model input data for all five analyzed countries, economic input data was country-specific due to differences in medical tariffs and direct medical resource utilization. We applied a hypothetical premium of € 2,000 (£ 1,665) to patients allocated to additional optimization procedures (related to echo-based optimization either manual or automatic). When conducting the economic model analysis from the perspective of the healthcare payer, favorable cost-effectiveness results were obtained for the CRT group with SO *vs*. NSO, in all five countries. ICERs at the 1 year follow-up were found to range from € 22,226 (Spain) to € 26,977 (Italy) per QALY gained. This implies that already after one year, ICERs are well below the commonly accepted willingness-to-pay threshold of € 30,000 (£ 25,000) determining cost-effectiveness. Because of savings in the systematically optimized group attributable to avoided HF hospitalizations over one year, the assumed premium for systematic optimization can be partially offset in subsequent years. Cost savings accrue during subsequent follow-up time periods so that this method becomes even more cost effective after 1 year in all countries. These favorable preliminary economic outcomes were confirmed by results from the sensitivity analysis. The savings in HF hospitalization costs predicted by the model are consistent with the results from the abstract published by Tarab and co-workers for adaptive CRT in the US [25]. The average hospitalization reduction per year (normalized by HF hospitalization cost to neutralize differences between countries) is close to 12 % in our model as compared with 10 % for the Adaptive CRT device (Medtronic, Minneapolis, MN, USA).

According to these preliminary findings, systematic CRT optimization represents integral part of the long-term management of CRT patients. However, CRT optimization with traditional echocardiography methods is time consuming and inadequately reimbursed in most countries, resulting in a suboptimal optimization management of CRT recipients. Devices with frequent and systematic reprogramming features might therefore be excellent tools which can aid to alleviate the time burden for follow-up device optimization performed by medical specialists treating CRT patients.

Our exploratory economic model analysis has a number of limitations. Due to a shortage of long-term clinical study data, we had to use assumptions on the probability of HF hospitalizations and survival in both groups for the follow-up time periods exceeding one year. We also restricted the time horizon of the analysis to five years although a sizable proportion of patients with systematic CRT optimization are predicted to be alive after this time and who could further benefit from the sustained effects delivered by systematic optimization. Additionally, health state utilities directly derived from the CLEAR post-hoc study were not available. Therefore, our assumptions require confirmation by further randomized long-term studies.

Based on the promising cost-effectiveness results from our exploratory economic analysis, systematic CRT optimization should be given more attention in the follow-up routine management of device recipients. However, time restrictions for CRT-specialists will most likely remain to exist for the present and will challenge a rapid adoption of this apparently beneficial method. Programming of systematic and frequent CRT optimization through implanted devices might be the future to at least partially resolve these issues.

## Conclusions

A longitudinal economic cohort model was developed to assess the cost-effectiveness of systematic CRT optimization (3 times a year) vs. non-systematic CRT optimization (less than 3 times a year), whatever the method used (manual echo or device-based using SonR®) in five European countries. The model predicted an incremental cost-effectiveness ratio (ICER) ranging between € 22,226 for Spain and € 26,977 for Italy at the 1-year follow-up. Employing a willingness-to-pay threshold of € 30,000 per QALY gained, the SO method developed into a cost-effective strategy from one year onwards. An exploratory analysis on CRT-D optimization showed that SO could improve cost-effectiveness by 27 % to 30 % (SO CRT-D vs. optimal pharmacological treatment alone and standard CRT-D vs. optimal treatment, respectively) at 5 years of follow-up, depending on the country analyzed.

## Additional file

Additional file 1:
**Table A.** Model assumptions for CRT optimization schedules. **Table B.** Assumptions for sensitivity analysis (example for Germany).
