# Supplementary material for: Exploratory cost-effectiveness analysis of cardiac resynchronization therapy with systematic device optimization vs. standard (non-systematic) optimization: a multinational economic evaluation
Source: Health Econ Rev. 2015 Jul 11;5:19. doi: 10.1186/s13561-015-0057-3 (PMC4498000; doi:10.1186/s13561-015-0057-3)
Supplement: Additional file 1: — Table A. Model assumptions for CRT optimization schedules. Table B. Assumptions for sensitivity analysis (example for Germany). [file 13561_2015_57_MOESM1_ESM.doc]

**Additional file 1**

**Table A** Model assumptions for CRT optimization schedules

Model variable Before Month 3 Month 6 Year 1 Year 2-5

discharge visit visit visit visits * Source

Systematic CRT optimization

Consultation + auto optimization 100% 100% 100% 100% 100%

Standard CRT optimization

Consultation only 20% 48% 77% 100% 100%

Consultation + echo optimization 80% 52% 23% 0% 0%

* Quarterly visits are assumed for each year

**Table B** Assumptions for sensitivity analysis (example for Germany)

Model variable Base case Low High Distribution Source

Estimate estimate estimate estimate

Risk reduction for all-cause mortality

(systematic *vs*. standard optimization group) ^a^ -52% -0% -60% Beta , assumption

Risk reduction for HF hospitalization

(systematic *vs*. standard optimization group) ^a^ -48% -36% -60% Beta , assumption

Utility scores (EQ-5D)

NYHA class I 0.815 0.781 0.850 Beta

NYHA class II 0.720 0.693 0.749 Beta

NYHA class III 0.590 0.551 0.629 Beta

NYHA class IV 0.508 0.412 0.605 Beta

Premium for automatic sensor lead ^b^ € 2,000 € 1,000 € 3,000 Triangular Assumption

Monthly drug costs € 59 € 30 € 89 Triangular Assumption

^a^ Base case risk reduction value based on clinical outcomes at 12 months

^b^ This cost has been taken into account for the systematic optimization group only
